# Elucidating the multiscale mechanisms and therapeutic targets of caffeic acid in gastric cancer: a synergy of computational and experimental approaches

**DOI:** 10.3389/fmolb.2025.1663517

**Published:** 2025-10-01

**Authors:** Hongrong Zhang, Yufan Li, Ya Li

**Affiliations:** ^1^ School of Medical Informatics Engineering, Anhui University of Chinese Medicine, Hefei, Anhui, China; ^2^ The First Affiliated Hospital, Anhui University of Chinese Medicine, Hefei, Anhui, China

**Keywords:** caffeic acid, gastric cancer, machine learning, molecular dynamics simulations, experimental verification

## Abstract

**Introduction:**

Gastric cancer is a malignant tumor with high incidence and mortality rates worldwide, and effective therapeutic strategies targeting its complex pathological processes are limited. Caffeic acid is a phenolic compound derived from natural plants and has attracted attention for its potential anticancer properties; however, its mechanism of action in gastric cancer has not been fully elucidated.

**Methods:**

In this study, a multimodal computational framework integrating multiomics, machine learning, and molecular dynamics simulations, combined with *in vitro* experiments, was used to systematically investigate the molecular mechanism of caffeic acid against gastric cancer.

**Results:**

Among the predicted targets, FZD2—a major receptor that mediates noncanonical WNT/Ca2+ signaling—was identified as a core regulatory hub associated with tumor progression and metastasis. Molecular dynamics simulations further revealed a stable binding interaction between caffeic acid and FZD2. An in vitro EMT model was established by treating human gastric cancer cells with TGF-β1. The results showed that caffeic acid intervention inhibited cell migration, invasion, and EMT progression while reducing FZD2 protein expression.

**Discussion:**

This study confirmed that caffeic acid regulates FZD2 expression and inhibits the activation of the noncanonical Wnt5a/Ca^2+^/NFAT signaling pathway, thereby interfering with gastric cancer–related pathological processes. These findings reveal the molecular mechanism of caffeic acid in gastric cancer and reflect the value of natural products in cancer research.

## 1 Introduction

Gastric cancer (GC) is the fifth most common malignancy worldwide and a leading cause of cancer-related death (Mousavi et al., 2025). Recent studies have reported more than one million new diagnoses of GC and approximately 769,000 deaths annually. It is primarily observed in Eastern Europe and Eastern Asia (Christodoulidis et al., 2024). Additionally, the incidence of GC has been reported to be increasing among younger individuals (Tan et al., 2024). Currently, the main treatments for GC are surgical resection and targeted drug therapy. Surgery aims to remove all visible tumors; however, microscopic residual tumor cells may persist, which can contribute to the recurrence of GC. Although trastuzumab, a monoclonal antibody that targets HER2, has demonstrated clinical efficacy in patients with HER2-positive GC, drug resistance limits its effectiveness (Zhang et al., 2025; Sabbagh et al., 2025). Trastuzumab deruxtecan, an antibody‒drug conjugate, has demonstrated greater efficacy than trastuzumab; however, the risk of side effects such as anemia, neutropenia, and lung disease, including pneumonitis, limits its clinical use (Shitara, 2024; Yamaguchi et al., 2023). Therefore, reducing the mortality rate and incidence of GC and finding safer, more effective drugs for its treatment have become essential.

Natural herbs have shown promising advantages in cancer therapy, offering high efficacy with relatively low toxicity and fewer adverse effects. They are currently under active investigation in biochemistry and pharmacology as potential sources of complementary and alternative anticancer agents (Xi et al., 2025). Cornus officinalis, a widely distributed medicinal herb, has long been used in traditional medicine. It has multiple pharmacological effects, including anti-inflammatory, antioxidant, and immunomodulatory activities (Badoni et al., 2024). Several bioactive compounds isolated from Cornus officinalis, such as loganic acid, quercetin, and secoxyloganin, have demonstrated anticancer effects on various malignancies by modulating apoptosis, metastasis, and inflammatory pathways (Kim et al., 2023; Feng et al., 2024; Ma and Peng, 2024). Previous studies have shown that Cornus officinalis contains loganic acid, quercetin, and secoxyloganin, as well as related phenolic acids and anthocyanins, including caffeic acid (CA) and pelargonidin (Czerwinska et al., 2018). CA, a phenylpropanoid polyphenol, has exhibited therapeutic potential in cancer treatment (Pavlikova, 2022; Alam et al., 2022). It has been shown to inhibit the proliferation of triple-negative breast cancer cells and tumor stem cells and to reduce the expression of CD44, a tumor stem cell marker, which may contribute to its antitumor activity (Xie et al., 2024). In addition, Yin et al. reported that CA inhibited the proliferation of prostate cancer cells (PC-3 and LNCaP) and induced reactive oxygen species (ROS), cell cycle arrest, and apoptosis in a concentration-dependent manner (Yin et al., 2024). Although CA exhibits promising anticancer properties, its mode of action in GC remains to be elucidated through further research.

Multiomics integrates high-throughput technologies such as genomics, transcriptomics, proteomics, and metabolomics to provide a comprehensive understanding of molecular complexity and its relevance to health and disease (Alemu et al., 2025). Machine learning (ML) uses algorithms to analyze large datasets, identify hidden patterns, and make predictions, showing great promise in medical research, including applications in disease diagnosis and herbal medicine discovery (Lim et al., 2024). Molecular dynamics (MD) simulations, which are based on Newtonian mechanics, enable atom-level exploration of molecular structure, motion, and thermodynamic properties. With advances in computing and algorithms, MD has become a powerful tool complementing experimental and theoretical studies (Yu et al., 2024).

**FIGURE 1:**
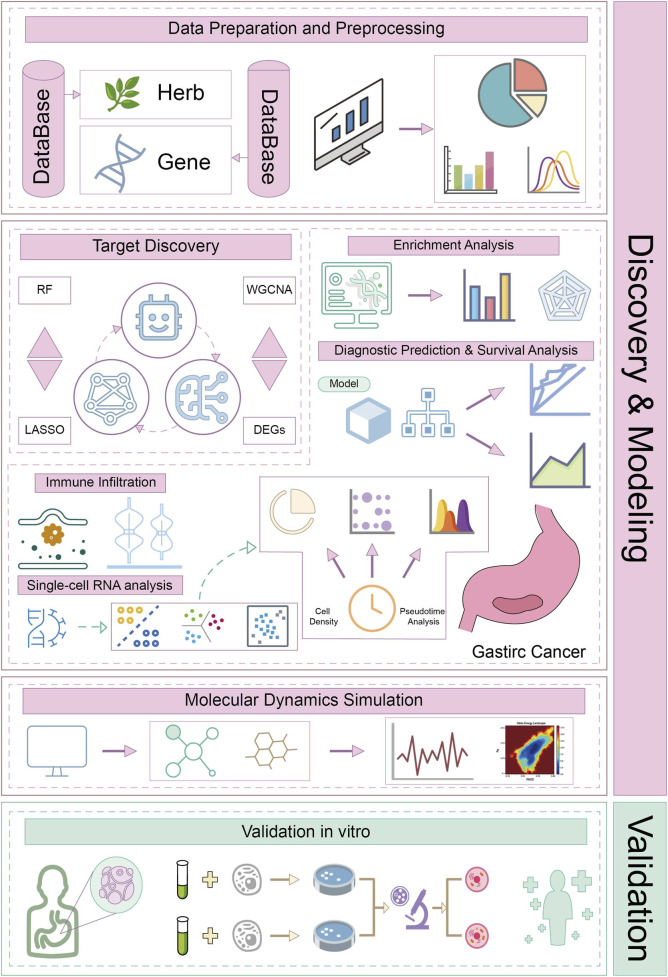
Workflow of this study.

To investigate the potential mechanism of action of CA against GC, this study used a combination of multiomics techniques, machine learning, and molecular dynamics simulations to screen and analyze key targets and pathways. In addition, *in vitro* experiments were conducted to further corroborate our findings (Figure 1). Furthermore, this research provides a theoretical and experimental basis for the use of CA in GC treatment and offers new insights into modern pharmacological studies.

## 2 Materials and methods

### 2.1 Data collection

A total of 448 GC clinical samples, comprising 412 tumor samples and 36 normal samples, were obtained from the TCGA database for the purpose of screening for biomarkers predictive of GC. Concurrently, the single-cell RNA sequencing dataset GSE163558, and four microarray datasets, GSE65801, GSE13911, GSE29272, and GSE79973, were retrieved from the GEO database. GSE163558 comprised three primary GC cases, one normal tissue sample, and six metastatic GC cases. The four microarray datasets were composed as follows. GSE65801 included 32 normal and 32 GC samples. GSE13911 contained 31 normal and 38 GC samples. GSE29272 provided 134 normal and 134 GC samples. GSE79973 consisted of 10 normal and 10 GC samples. Furthermore, the components of the herbal medicine Cornus officinalis were collated using the HERB database. The HERB database contains data of 7,263 distinct herbal medicines and 49,258 individual herbal components (Zhai et al., 2025). A total of 278 components of Cornus officinalis were identified. The PubChem database was subsequently used to standardize and download the SDF structure data.

### 2.2 Screening of potential hub genes

To identify hub genes in GC, four algorithmic models were constructed to analyze the clinical samples. Initially, the limma package in R was used to normalize the 448 clinical samples. Differentially expressed genes (DEGs) were selected using thresholds of |log2FC| > 2 and FDR <0.001, thereby generating gene cohort A. Subsequently, the clinical samples were clustered using the WGCNA algorithm, with a soft threshold of β = 8, to construct the gene coexpression network. The topological overlap matrix (TOM) was used for hierarchical clustering, and the Pearson correlation coefficient was used to evaluate the relationship between each module and the GC. The module with the highest correlation was identified as the core module, and its genes were included in gene cohort B. Furthermore, a random forest (RF) model was used to classify genes in the clinical samples via decision trees. The genes were randomly assigned, with n_tree = 1,000 and 10-fold cross-validation as the default parameters. The genes with the highest feature importance were selected and compiled into gene cohort C. Subsequently, the least absolute shrinkage and selection operator (LASSO) regression algorithm was applied to analyze the genes of the clinical samples. To ascertain the optimal parameters, a 10-fold cross-validation was conducted, resulting in the compilation of a core hub gene set for GC as gene cohort D.

### 2.3 Gene functional enrichment analysis

To explore the regulatory roles of hub genes in the pathogenesis of GC, we initially used the Venn diagram package to identify the intersecting hub genes among gene cohorts A, B, C, and D. Gene Ontology (GO) enrichment analysis was used to classify genes on the basis of functional similarities, thereby revealing potential biological processes. GO enrichment analysis comprises three ontologies: biological process (BP), cellular component (CC), and molecular function (MF). Kyoto Encyclopedia of Genes and Genomes (KEGG) enrichment analysis was used to identify the pathways in which genes were significantly enriched, revealing the molecular mechanisms and signaling pathways involved in gene expression changes. The intersecting hub genes were subjected to GO and KEGG enrichment analyses, with a significance threshold of P < 0.05, and the results were visualized using the ggplot2 package.

### 2.4 Prognostic value assessment and diagnostic model construction

To further investigate the prognostic value of the intersecting hub genes in GC, a survival analysis was performed using the survival R package, with a P value <0.05 indicating prognostic significance. The intersecting hub genes that exhibited prognostic value were designated core genes, and survival curves were plotted for these genes. Moreover, to evaluate the diagnostic potential of the core genes in GC, diagnostic models have been developed. Receiver operating characteristic (ROC) curves were used to quantify the diagnostic accuracy of these core genes for GC, with the x-axis representing the false-positive rate and the y-axis representing the true positive rate. Area under the curve (AUC) was used to assess the diagnostic efficacy of the models. A higher AUC value is indicative of enhanced diagnostic capability for GC and greater accuracy.

### 2.5 Immune infiltration analysis in TME

To investigate the infiltration levels and correlations between core genes and immune cell characteristics in the tumor microenvironment (TME), we used the Estimation of Stromal and Immune Cells in Malignant Tumor Tissues Using Expression Data (ESTIMATE) algorithm and the Cell Type Identification by Estimating Relative Subsets of RNA Transcripts (CIBERSORT) algorithm to calculate the stromal score and immune score, estimate the score, and assess the correlations between core genes and 22 types of immune cells in GC. The results obtained from the ESTIMATE algorithm may prove useful in clinical decision-making, as tumors with higher immune scores are more likely to respond favorably to immunotherapy. Furthermore, the CIBERSORT algorithm, which is based on linear support vector regression, allows for the calculation of the relative abundance of 22 immune cell subtypes by analyzing core gene expression data. This enables the characteristics of the tumor immune system to be elucidated and facilitates the prediction of the prognosis of GC. A visual representation was subsequently generated using the ggpubr R package.

### 2.6 scRNA-seq analysis

To increase the precision of the data analysis, we undertook a single-cell analysis utilizing the sequencing dataset GSE163558. Following preprocessing, the data were filtered using the Seurat R package. The filtering criteria included gene counts per cell between 500 and 6,000, a mitochondrial content less than 25%, and the removal of the smallest and largest 1% of cells based on the rRNA expression proportion. To better conform to the assumption of a normal distribution, the NormalizeData function was used to transform the expression values using the LogNormalize method, with the aim of minimizing intercellular variation. The top 2000 highly variable genes were identified using the FindVariableFeatures function, after which the data were further scaled. Furthermore, we conducted dimensionality reduction using the “RunPCA” function and corrected batch effects with the Harmony algorithm. We then clustered the cells using graph-based clustering algorithms. A K-Nearest Neighbours (KNN) clustering graph was constructed in PCA space using Euclidean distance, cell-to-cell edge weights were optimized using Jaccard similarity, and clustering was refined using the Louvain algorithm. This was followed by cell grouping with the “FindNeighbors” and “FindClusters” functions. Furthermore, cell cluster types were annotated by referencing the Human Primary Cell Atlas dataset, and the expression levels and patterns of core genes across different cell types were examined. Finally, Pseudo-Temporal Analysis was conducted using Monocle to describe the trajectory of the cell states based on the similarity of the cellular transcriptional profiles and to plot the cell density along the timeline. Additionally, the changes in the expression of core genes over time were investigated.

### 2.7 Virtual screening and molecular dynamics simulations

To gain further insight into the molecular mechanism of Cornus officinalis in the treatment of GC, this study was conducted using large-scale virtual screening and MD. The protein structures of CINP (id:8cih), FZD2 (id:7x8p), GABARAPL2 (id:7lk3), GRB10 (id:1nrv), ROCK1 (id:7s26), and SLFN11 (id:7zel) were retrieved from the RCSB database. The remaining 12 protein structures were predicted using AlphaFold 3. In addition, the small-molecule SDF structures from the PubChem database were converted into MOL2 format using Open Babel. An oral bioavailability model was subsequently constructed in accordance with Lipinski’s rule of five and Veber’s rules. Small molecules that satisfied both criteria were deemed to exhibit favorable oral bioavailability. The small molecules that satisfied both criteria were subjected to large-scale virtual screening against the retrieved PDB structures. Binding energies of less than −7 kcal·mol-1 were deemed indicative of a strong binding capacity, and the proteins that exhibited the highest binding affinity for the smallest molecules were selected for subsequent analyses. Furthermore, toxicity prediction models were constructed using the KNN, Multilayer Perceptron (MLP), Support Vector Machine (SVM), and RF algorithms. The models evaluated the following properties: mutagenicity, carcinogenicity, estrogenicity, androgenicity, skin irritation, and acute oral toxicity. A toxicity score greater than 50 was considered indicative of toxicity, with higher scores reflecting increased toxicity. Nontoxic small molecules were subsequently selected for MD simulations using Gromacs software. The Amber force field was used for biomacromolecules, whereas the GAFF force field was used for small molecules. These were positioned within a defined cubic box with periodic boundary conditions and water solvation. The particle mesh Ewald (PME) method was subsequently used to conduct equilibrium simulations under both NPT and NVT conditions. Furthermore, the steepest descent algorithm was used to minimize the system energy, with a simulation time frame of 100 ns. Free energy profiles were plotted to identify energy wells with the lowest binding energies and their corresponding frame numbers, and the molecular conformations at these frames were visualized using PyMOL.

### 2.8 Validation of FZD2 expression in public datasets

To validate the expression of FZD2 in GC samples, four datasets from the GEO database (GSE65801, GSE13911, GSE29272, and GSE79973) were used. Differential expression analysis was conducted using the limma package in the R environment. Prior to analysis, all raw expression data were normalized. The significance thresholds were set at a P value <0.05 and |log2FC| > 2. To increase statistical power, the GSE29272 and GSE79973 datasets were combined. Furthermore, the expression of FZD2 was investigated on two additional online platforms, TIMER 2.0 and GEPIA 2, to validate its expression pattern.

### 2.9 Culture, modeling, and grouping of GC cell lines

The human GC cell line MKN-45 (CL-0292) and NCI-N87(CL-0169) were obtained from Wuhan Prioella Biotechnology Co., Ltd. The cells were cultured in RPMI-1640 medium supplemented with 10% fetal bovine serum (FBS) and 1% penicillin‒streptomycin at 37 °C in a humidified atmosphere containing 5% CO_2_. Cells in the logarithmic growth phase were used for subsequent experiments. To induce epithelial‒mesenchymal transition (EMT), the cells were exposed to 10 ng/mL TGF-β1 for 24 h, as previously described (Zhang et al., 2020). After EMT induction with TGF-β1, the cells were divided into three groups: the EMT model group (M), the low-dose caffeic acid group (4 μM) (CA-L), and the high-dose caffeic acid group (8 μM) (CA-H). CA was dissolved in DMSO to prepare an 8 mM stock solution and stored at −20 °C for later use. For treatment, the CA stock was diluted in culture medium to 8 μM and 4 μM (final DMSO <0.1%), the corresponding concentrations of CA were added to the CA-L/H groups, and the cells were further cultured for 24 h. The control group (C) was cultured in RPMI-1640 complete medium without any intervention. All groups were maintained under identical culture conditions. The adenovirus vector pADV-mCMV-EGFP-3×FLAG was used for FZD2 overexpression in the OE-FZD2 group. Specifically, the OE-FZD2 adenovirus (1 × 109 PFU/mL, 10 μL) was added to the cells in the induced EMT model group for 3 h. The open reading frame of FZD2 was cloned into the multiple cloning site downstream of the mCMV promoter in pADV-mCMV-EGFP-3×FLAG to construct adenovirus-OE-FZD2. The primers used were FZD2 ORF-F (5′-GGGGGGCGGCGGCCAGCATG-3′) and FZD2 ORF-R (5′-GCATACTTGTATTCAAACTA-3′).

### 2.10 Cell viability assay

MKN-45 cells in the logarithmic growth phase were digested, resuspended, and seeded into 96-well plates at a density of 6,000 cells per well. After 24 h of culture to allow cell adhesion, the cells were divided into a control group (0 μM) and CA groups (1/2/4/8/16/32 μM), with three independent replicates per group. The cells were treated for 24 h according to the experimental groups, and the medium in each well was discarded. Then, 10 μL of CCK8 solution and 100 μL of culture medium were added to each well. After incubation for 2 h, the absorbance at 450 nm was measured with a microplate reader, and the cell viability was recorded and calculated.

### 2.11 Transwell invasion assay

BD Matrigel was diluted with MEM at a ratio of 1:3, and 50 μL of the mixture was added to the upper chamber of the Transwell insert and incubated in an incubator for solidification. MKN-45 cells were digested and resuspended in serum-free medium to a final concentration of 1.25 × 10^4^ cells/mL. After thorough mixing, 200 μL of the cell suspension was added to the upper chamber of the Transwell insert. Then, 500 μL of complete medium was added to the lower chamber. The inserts were placed into the wells and incubated at 37 °C for 24 h. After incubation, the cells were stained with crystal violet for 10 min, excess stain was removed by washing, and cell invasion was observed and photographed using an inverted microscope.

### 2.12 Wound healing assay to evaluate cell migration

MKN-45 cells in the logarithmic growth phase were digested with trypsin and evenly seeded into six-well plates. Once the cells reached approximately 90% confluence, a straight scratch was made in each well using a sterile pipette tip guided by a sterile ruler. The cells were then treated according to the experimental groups for 24 h. Photographs were obtained using an inverted microscope at 0 h and 24 h to observe wound closure and cell migration.

### 2.13 Immunofluorescence detection of intracellular Ca^2+^ levels

MKN-45 cells were digested, resuspended, and seeded into 12-well plates at a density of 2 × 10^5^ cells/mL. After cell adherence, treatments were applied according to the experimental design. Rhod-2 a.m. red was diluted with HBSS to a final concentration of 2 μM. Following 24 h of drug treatment, the culture medium was removed and replaced with 1 mL of diluted Rhod-2 a.m. red solution per well. The cells were incubated at 37 °C in 5% CO_2_ for 30 min and washed three times with serum-free medium to remove excess probe. Fluorescence images were captured under a fluorescence microscope. Images were acquired under identical exposure settings, and fluorescence intensity was quantified using ImageJ.

### 2.14 Western blot Analysis of EMT markers and key proteins in the Wnt5a/Ca^2+^/NFAT signaling pathway

Total protein was extracted from each group of cells. The protein concentration was determined using the BCA assay. The samples were prepared by adding 5× loading buffer and boiling at 100 °C for 10 min. Equal amounts of protein were loaded for electrophoresis, followed by transfer to membranes and blocking. The membranes were incubated overnight at 4 °C with primary antibodies against E-cadherin (1:10,000), N-cadherin (1:10,000), vimentin (1:10,000), Snail (1:1,000), Wnt5a (1:2,000), CaN (1:5,000), FZD2 (1:1,000), NFAT1 (1:1,000), p-NFAT1 (1:2,000), PKC (1:10,000), and GAPDH (1:10,000) as internal controls. After washing, the membranes were incubated at room temperature for 30 min with appropriately diluted HRP-conjugated secondary antibodies: goat anti-rabbit (1:50,000) and goat anti-mouse (1:50,000). The protein bands were visualized using ECL substrate and analyzed for grayscale intensity using ImageJ software.

### 2.15 Co-immunoprecipitation

Cells were washed once with PBS and lysed in 500 μL of IP lysis buffer. The clarified lysates were divided into two equal portions and incubated overnight at 4 °C with either 2 μg of anti-Wnt5a antibody or 2 μg of rabbit IgG under gentle rotation. Subsequently, 100 μL of Protein A/G Agarose beads were added and the mixtures were incubated for 2 h at 4 °C, followed by four washes to remove non-specific binders. Immunocomplexes were resuspended in 100 μL of protein loading buffer and heated at 100 °C for 5 min. The precipitated proteins were then analyzed by immunoblotting.

### 2.16 Statistical analysis

All computational procedures were implemented using R (version 4.3.0) and Python (version 3.8). The data were analyzed using SPSS version 26.0. The distribution of the data was assessed using the Shapiro–Wilk test. The experimental results are expressed as the means ± standard deviations. The homogeneity of variance among groups was verified using Bartlett’s test. Group differences were assessed using ANOVA, and Tukey’s test was used for multiple comparisons. For pairwise comparisons, independent sample t tests were performed. A two-tailed p value <0.05 was statistically significant. Graphical representations of the *in vitro* data were generated using GraphPad Prism version 9.0.0.

## 3 Results

### 3.1 Selection of potential hub genes using ML algorithms

**FIGURE 2:**
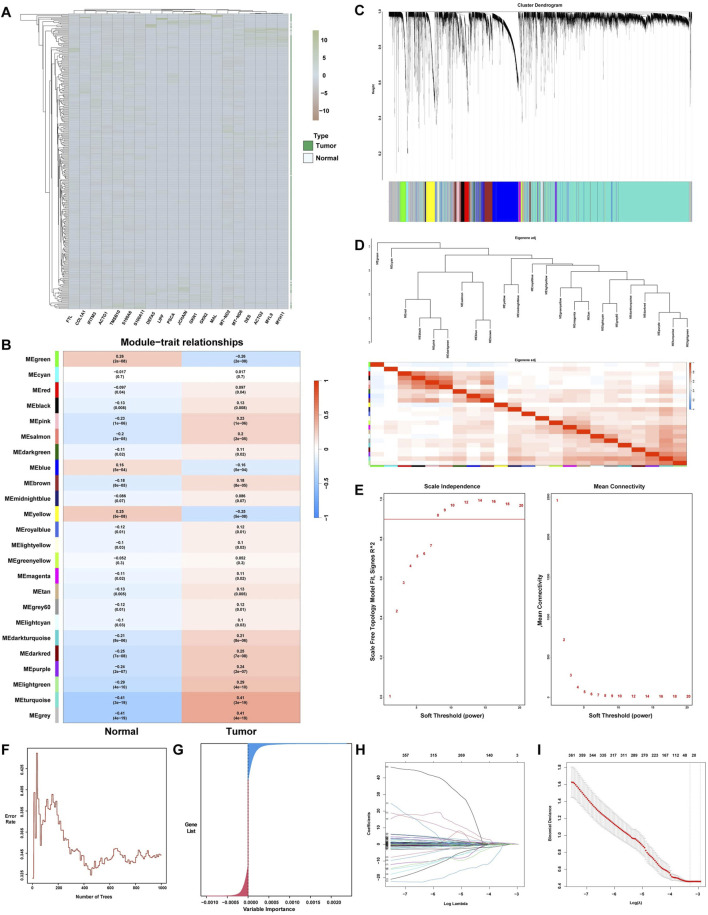
ML for the selection of potential hub genes in GC. **(A)** Complex heatmap of differential gene expression levels between the GC group and the normal group. **(B)** Heatmap displaying the characteristic genes of different ME modules associated with GC, with red and blue indicating positive and negative correlations between the modules and samples, respectively. **(C)** Gene clustering dendrogram based on the dissimilarity measure (1-TOM). **(D)** Clustering dendrogram of ME modules. **(E)** Analysis of the scale-free topology fit index for different soft-thresholding powers (β). **(F)** Error rates of 10-fold cross-validated random forest models and **(G)** Distribution of gene importance. **(H)** Lasso feature coefficient plot. **(I)** Validation of the LASSO regression model.

To establish the relationship between clinical sample data and hub genes, four algorithmic models were used to analyze the clinical sample dataset. Gene cohort A included 8,068 genes, of which 7,665 are upregulated and 403 are downregulated. Figure 2A illustrates the 20 genes with the highest correlation coefficients. Subsequently, gene cohort B was subjected to analysis using the WGCNA algorithm. The adjacency matrix was transformed into a TOM using the optimal soft threshold and weighted by the Pearson correlation coefficient, thereby achieving a scale-free network distribution (Figures 2C,E). In addition, the Pearson correlation coefficient was used to assess analogous expression patterns, thereby facilitating the construction of a hierarchical clustering tree for the genes in question. Figure 2B and D illustrate the identification of 24 distinct modules. Notably, the module with the highest correlation was MEturquoise (r = 0.41). A total of 7,393 genes within the MEturquoise module were incorporated into gene cohort B. The RF algorithm was used to construct decision trees, identifying 5,656 genes with a variable importance score greater than zero, which were subsequently compiled into gene cohort C (Figures 2F,G). Finally, gene cohort D was generated by eliminating noncore genes through the application of a LASSO algorithm model with 10-fold cross-validation. Gene cohort D included 10,237 genes (Figures 2H,I).

### 3.2 Functional enrichment analysis

**FIGURE 3:**
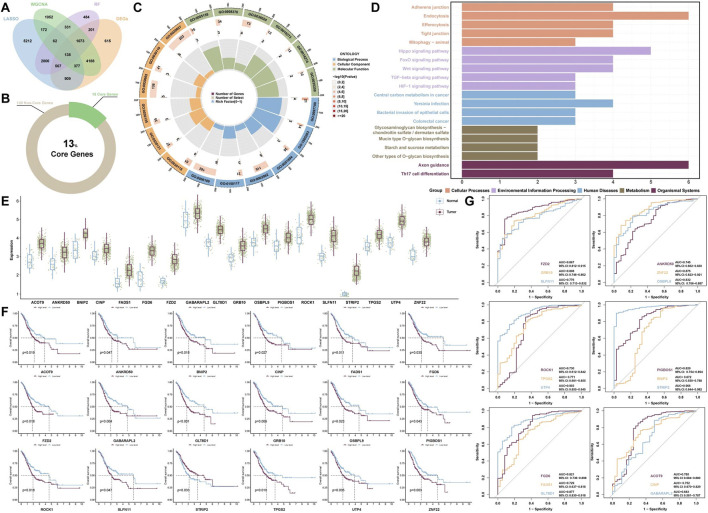
Functional enrichment analysis and predictive model construction. **(A)** Venn diagram displaying the intersection of the hub genes identified by the four algorithmic models. **(B)** Proportion of core genes among the intersecting hub genes. **(C)** GO enrichment analysis and **(D)** KEGG enrichment analysis of the intersecting hub genes. **(E)** Expression levels of core genes between the GC group and the normal group. **(F)** Survival prediction models for core genes and **(G)** ROC diagnostic prediction models.

To gain further insight into the biological mechanisms that drive GC progression, we identified 138 intersecting hub genes from gene cohorts A, B, C, and D (Figure 3A). GO enrichment analysis of 138 genes revealed significant relationships between biological behaviors such as acetylgalactosaminyltransferase activity (GO:0008376), arginine N-methyltransferase activity (GO:0016273), and nuclear glucocorticoid receptor binding (GO:0035259) and GC progression (Figure 3C). Furthermore, KEGG enrichment analysis indicated that pathways such as the Wnt signaling pathway, FoxO signaling pathway, and Hippo signaling pathway may contribute to the mechanisms underlying GC development (Figure 3D).

### 3.3 Prognostic analysis and diagnostic model evaluation

To assess the prognostic value of the intersecting hub genes, we performed a survival analysis. The analysis identified 18 core genes with prognostic significance: ACOT9, ANKRD50, BNIP2, CINP, FADS1, FGD6, FZD2, GABARAPL2, GLT8D1, GRB10, OSBPL9, PIGBOS1, ROCK1, SLFN11, STRIP2, TPGS2, UTP4, and ZNF22. The aforementioned genes are highly expressed in tumor tissues and expressed at low levels in normal tissues (Figures 3B,E). Additionally, the survival analysis results indicated that, with the exception of patients with high STRIP2 expression, patients in the low-expression group presented higher survival rates than did those in the high-expression group for the remaining core genes (Figure 3F).

Notably, the diagnostic models constructed using each of the core genes individually demonstrated high accuracy (Figure 3G). The top five diagnostic models, ranked by their performance, are as follows: STRIP2 (AUC = 0.966, 95% CI: 0.944–0.982), UTP4 (AUC = 0.903, 95% CI: 0.855–0.945), GLT8D1 (AUC = 0.877, 95% CI: 0.830–0.918), ZNF22 (AUC = 0.875, 95% CI: 0.823–0.921), and FZD2 (AUC = 0.867, 95% CI: 0.812–0.915). These findings indicate that these core genes exhibit strong diagnostic performance.

### 3.4 Core genes and immune landscape correlation

**FIGURE 4:**
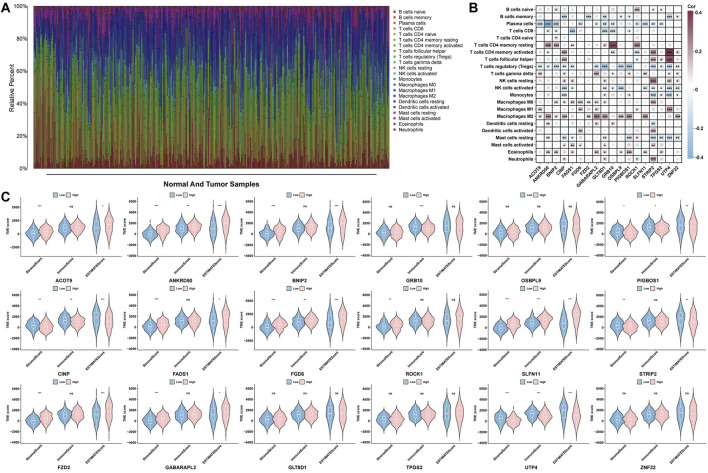
Analysis of the immune microenvironment. **(A)** Bar chart of immune cell populations in GC samples and normal samples. **(B)** Heatmap of the correlations between core genes and 22 immune cell types. **(C)** Violin plot depicting the TME scores of core genes across different samples.

Based on the ESTIMATE and CIBERSORT algorithms, we performed a correlation analysis between immune infiltration and core genes in the TME. The results from the CIBERSORT algorithm indicate that the majority of core genes are negatively correlated with T-cell regulatory (Tregs) and plasma cells and positively correlated with M2 macrophages (Figures 4A,B). Additionally, since the estimate score is derived from both the stromal and immune scores to estimate tumor purity, we focused on comparing the estimate scores. Figure 4C shows significant differences in the estimate scores for ACOT9, ANKRD50, BNIP2, PIGBOS1, CINP, FADS1, FGD6, SLFN11, STRIP2, FZD2, GABARAPL2, and UTP4 between the high- and low-risk expression groups. However, GRB10, OSBPL9, ROCK1, GLT8D1, TPGS2 and ZNF22 were not significantly correlated.

### 3.5 scRNA analysis results

**FIGURE 5:**
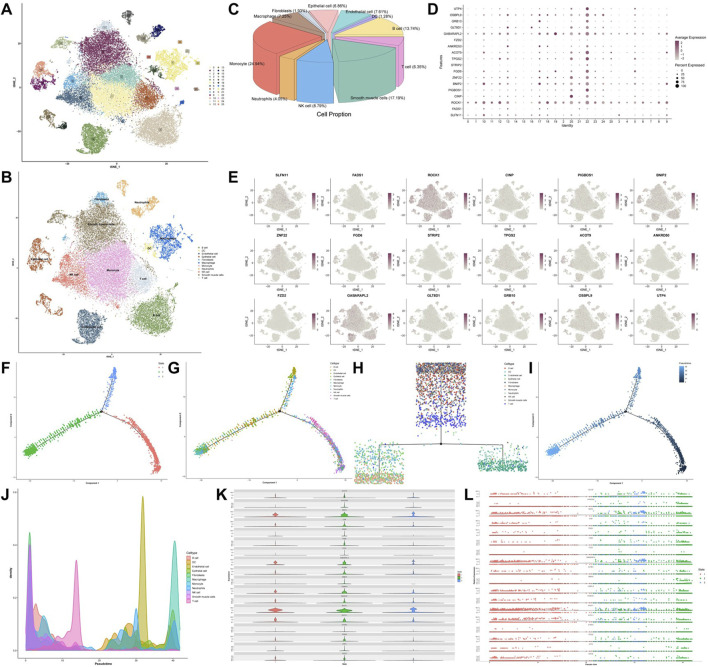
Single-cell transcriptome analysis atlas. **(A)** TSNE clustering analysis of single-cell samples. **(B)** Annotation of clustered cell types. **(C)** Pie chart showing the proportions of different cell types in single-cell samples. **(D)** Core gene expression across 26 clusters. **(E)** Distribution of core genes across 11 cell types. **(F–I)** Pseudotime trajectory plots of cellular development. **(J)** Pseudotime trajectory plot depicting the cell density along the developmental timeline. **(K)** Violin plots of core gene expression levels along the pseudotime trajectory and **(L)** scatter plots of relative expression levels.

To explore the expression profiles of 18 core genes across different cell types, we analyzed the scRNA-seq data. After stringent quality control and preprocessing, we selected a resolution of 1.0 for downstream analyses, as higher resolutions enable finer detection of gene expression patterns at the single-cell level. The analysis revealed 26 distinct subclusters (Figure 5A). Cell annotation revealed several types, including B cells, dendritic cells (DCs), endothelial cells, epithelial cells, fibroblasts, macrophages, monocytes, neutrophils, NK cells, smooth muscle cells, and T cells (Figures 5B,C). We then examined the expression levels of the core genes in different cell types and found that all the genes were expressed to some extent. ROCK1 and GABARAPL2 were broadly distributed, whereas FZD2 was highly expressed in epithelial cells (Figures 5D,E). In addition, pseudotime analysis of the scRNA-seq data revealed that T cells, smooth muscle cells, NK cells, and B cells were grouped into subset 1; DCs, epithelial cells, macrophages, and monocytes were grouped into subset 2; and endothelial cells, neutrophils, and fibroblasts were grouped into subset 3 (Figures 5F–I). Furthermore, pseudotime analysis revealed that the relative density of NK cells and fibroblasts decreased over time, whereas that of T cells, endothelial cells and macrophages progressively increased (Figure 5J). Finally, the expression levels of the core genes differed between pseudotime states, with most genes exhibiting markedly higher expression in state 2. In contrast, a few proteins, including BNIP2 and ROCK1, were more highly expressed in states one and 3 (Figures 5K,L).

### 3.6 Results of virtual screening and MD simulations

**FIGURE 6:**
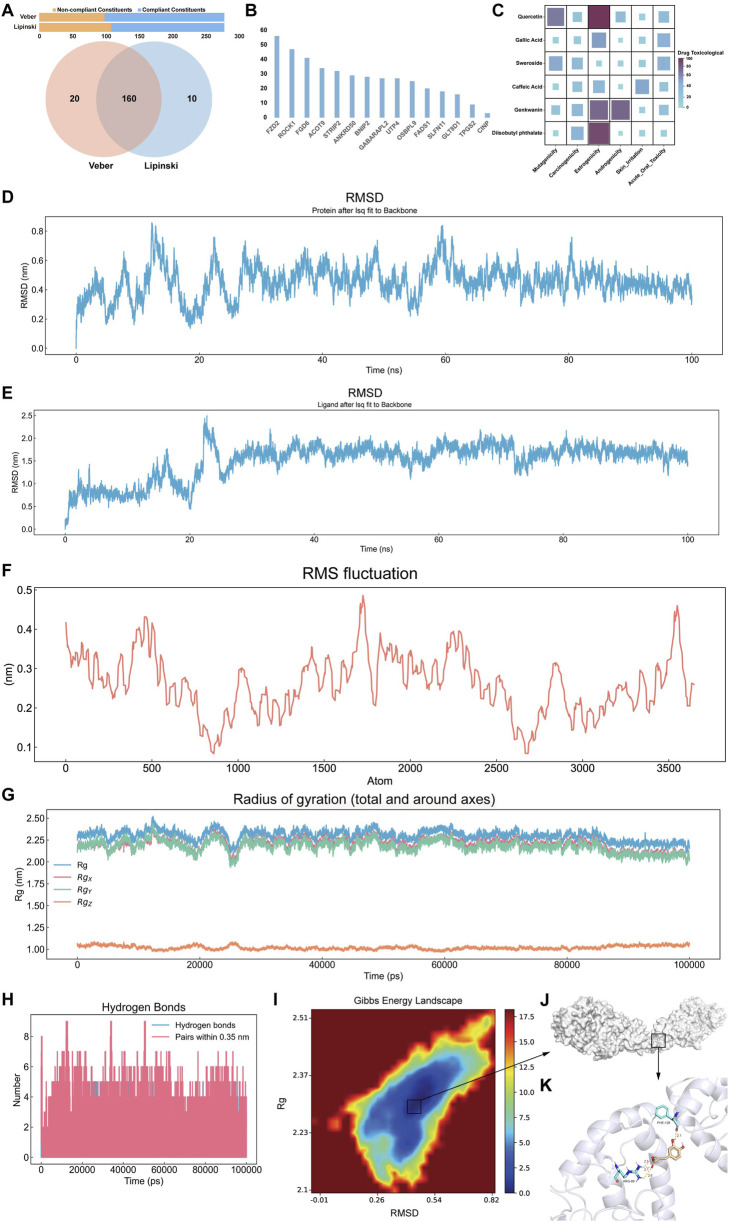
Pharmaceutical component evaluation, virtual screening, and MD. **(A)** Bar chart displaying the ingredients that satisfy Veber’s and Lipinski’s rules and a Venn diagram showing the effective ingredients that meet both oral bioavailability criteria. **(B)** Bar chart displaying the virtual screening results of compounds from effective ingredients against core gene targets, with the Y-axis indicating the number of compounds exhibiting strong binding affinity to the target protein. **(C)** Heatmap showing the toxicological prediction results of effective ingredients. **(D)** RMSD trajectory curve of the complex. **(E)** RMSD trajectory curve of the ligand in the complex. **(F)** RMSF trajectory curve of the complex. **(G)** Rg curve of the complex. **(H)** Number of hydrogen bonds in the complex. **(I)** Free energy landscape diagram of the complex. **(J)** The conformation with the lowest energy in the system and **(K)** its detailed diagram.

The oral bioavailability model revealed that 278 Cornus officinalis compounds, 170 of which met Lipinski’s rule of five, 180 met Veber’s rules, and 160 met both criteria (Figure 6A). Subsequently, 42,984 virtual screening simulations were performed between the 160 active compounds and 18 core genes. The results revealed that many Cornus officinalis compounds strongly bind to FZD2, suggesting a potential role in modulating FZD2 to influence GC progression (Figure 6B). We subsequently predicted the toxicity of the top six small molecules selected from the active ingredients of Cornus officinalis based on their binding energy to FZD2. Quercetin, gallic acid, genkwanin, and diisobutyl phthalate showed significant toxicity in the estrogenicity assays. In addition, the toxicity model indicated that both genkwanin and quercetin were toxic in the androgenicity and mutagenicity assays. Notably, sweroside and CA did not show toxicity in any of the toxicity prediction models (Figure 6C). CA was selected for subsequent molecular dynamics analysis. The root mean square deviation (RMSD) results revealed that the atomic fluctuations in the FZD2-CA complex stabilized after 40 ns, whereas the root mean square fluctuation (RMSF) indicated regions of high structural stability (Figures 6D,E). In addition, radius of gyration (Rg) and hydrogen bonding analyses revealed that the atomic distribution was more compact than the random distribution was and that the interactions within the complex were stronger (Figures 6G,H). Finally, the free energy landscape indicated the presence of a low-energy well between FZD2 and CA (Figure 6I). The corresponding frames from this energy well, visualized using PyMOL, revealed several hydrogen bond interactions, with FZD2 tightly surrounding the CA molecule (Figures 6J,K).

### 3.7 External validation of FZD2 expression

**FIGURE 7:**
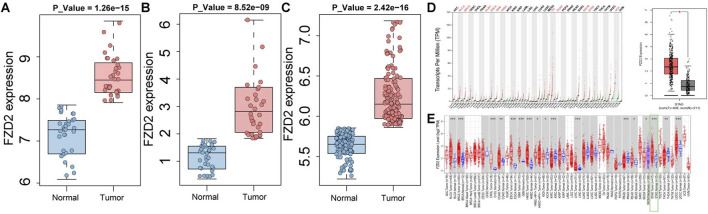
Validation of FZD2 in public datasets. **(A)** Analysis in the GSE65801 dataset. **(B)** Analysis in the GSE13911 dataset. **(C)** Analysis in a merged cohort combining GSE29272 and GSE79973. **(D)** GEPIA 2 analysis results. **(E)** TIMER 2.0 analysis results.

The validation of FZD2 expression using external datasets revealed a trend consistent with the initial findings. In these datasets, FZD2 expression was consistently and significantly higher in cancer tissues than in normal tissues (P < 0.001), a pattern observed in both individual and merged dataset analyses (Figures 7A–C). Furthermore, this finding was corroborated using online data platforms. Analysis in GEPIA 2 demonstrated significantly higher FZD2 expression in tumor tissues (P < 0.05) (Figure 7D). Similarly, analysis via the TIMER 2.0 platform also indicated that FZD2 was significantly upregulated in GC tissues (P < 0.001) (Figure 7E).

### 3.8 Effects of drug treatment on the viability of MKN-45 cells

**FIGURE 8:**
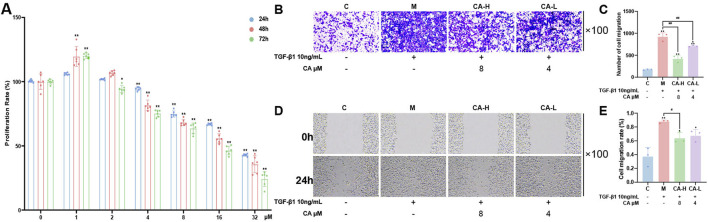
CA inhibits the migration and invasion of MKN-45 cells. **(A)** CCK-8 assay showing the effects of different concentrations of CA on cell viability (N = 6, per group). **(B,C)** Transwell assays assessing the effects of 8 μM and 4 μM CA on cell migration and quantitative analysis (N = 3, per group). **(D,E)** Wound healing assays evaluating the impact of CA on cell invasion (N = 3, per group). Compared with the group C, ^*^p < 0.05, ^**^p < 0.01. Compared with the group M, ^#^p < 0.05, ^##^p < 0.01. Compared with the group CA-H, ^▲^p < 0.05.

MKN-45 cells were treated with increasing concentrations of CA for 24,48 and 72 h. The results showed that CA inhibited MKN-45 cells in a dose-dependent manner within the concentration range of 4–32 μM, with cell proliferation rates decreasing as the concentration increased, and this inhibitory effect showed time dependency. Compared with 0 μM CA, 1 μM CA showed some promotion of GC cell proliferation, but the proliferation effect was not evident, whereas 2 μM CA had no significant effect on cell proliferation (Figure 8A). Considering the inhibitory and cytotoxic effects on cells, the 4 μM and 8 μM concentrations were selected as group CA-L and group CA-H intervention for 24h, respectively, for subsequent experiments.

### 3.9 Effects of drug treatment on the TGF-β1-induced invasion and migration of MKN-45 cells

After 24 h of TGF-β1 induction, compared with those in group C, some cells in group M exhibited a clear EMT phenotype, changing from an oval shape to a spindle-like shape. The number of cells invading through the lower chamber of the Transwell system significantly increased (Figures 8B,C), and the wound healing rate markedly increased (Figures 8D,E), with both differences being statistically significant (P < 0.01). Compared with those in group M, both group CA-L and group CA-H presented significantly fewer cells invading the lower chamber (P < 0.01) and markedly lower wound healing rates, with group CA-H showing a more pronounced effect (P < 0.05). The results are shown in Figures 8B–E.

### 3.10 Effect of drug intervention on intracellular Ca^2+^ levels in MKN-45 cells

**FIGURE 9:**
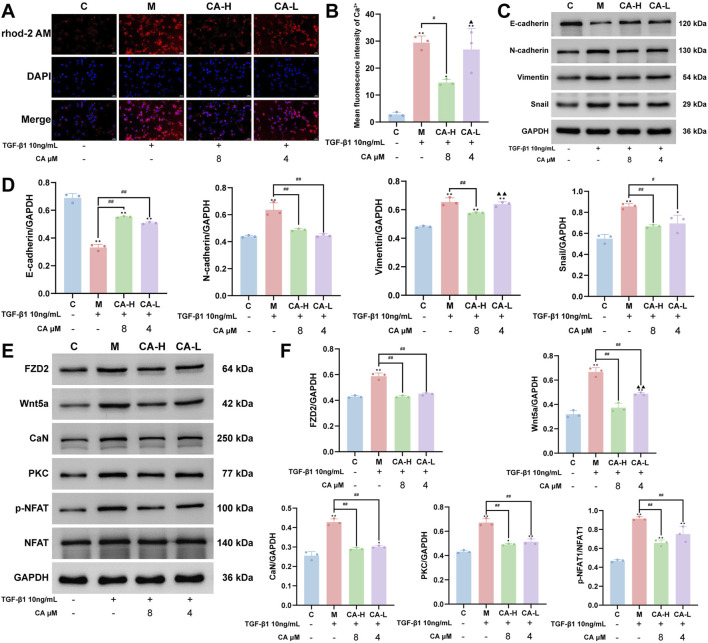
CA ameliorates the EMT phenotype of MKN-45 cells by suppressing FZD2 expression and the Wnt5a/Ca^2+^/NFAT signaling pathway. **(A,B)** Immunofluorescence analysis showing that CA treatment reduces intracellular Ca^2+^ levels in MKN-45 cells, along with the statistical analysis results (N = 3, per group). **(C,D)** Western blot analysis of the expression of EMT-related marker proteins after CA treatment and their quantitative analysis (N = 3, per group). **(E,F)** Western blot analysis of the regulatory effects of CA on FZD2 and key proteins in the Wnt5a/Ca^2+^/NFAT signaling pathway, along with the statistical analysis results (N = 3, per group). Compared with the group C, ^*^p < 0.05, ^**^p < 0.01. Compared with the group M, ^#^p < 0.05, ^##^p < 0.01. Compared with the group CA-H, ^▲^p < 0.05, ^▲▲^p < 0.01.

As shown in Figures 9A,B, compared with those in group C, the intracellular Ca^2+^ levels were significantly elevated in group M (P < 0.01). Compared with group M, group CA-H treatment effectively reduced the Ca^2+^ level (P < 0.05), whereas no significant effects were observed in group CA-L (P > 0.05). Furthermore, compared with group CA-H the Ca^2+^ level remained relatively elevated in group CA-L (P < 0.05).

### 3.11 Effects of drug treatment on EMT marker protein expression in MKN-45 cells

Compared with that in group C, the expression of E-cadherin was significantly lower and the expressions of N-cadherin, vimentin, and Snail were significantly greater in group M (P < 0.01). Compared with group M, both group CA-L and group CA-H treatments presented significantly upregulated E-cadherin (P < 0.01) and downregulated N-cadherin and Snail expressions (P < 0.05), with group CA-H exhibiting a more pronounced effect. Moreover, only group CA-H had significantly lower vimentin expression than group M did (P < 0.01), and its effect was significantly greater than that of the group CA-L (P < 0.01) (Figures 9C,D).

### 3.12 Effects of drug treatment on the expression of proteins in the Wnt5a/ca^2+^/NFAT signaling pathway in MKN-45 cells

Compared with those in group C, the expression levels of Wnt5a, CaN, FZD2, and PKC, as well as the relative ratio of p-NFAT to NFAT, were significantly greater in group M (P < 0.01). Compared with those in group M, both group CA-L and group CA-H presented significantly lower expression of Wnt5a, CaN, FZD2, and PKC and a lower p-NFAT/NFAT ratio (P < 0.01). Compared with group CA-H, group CA-L showed weaker inhibition of only Wnt5a expression (P < 0.01), suggesting a dose-dependent effect, while no significant difference in the other proteins was detected between the two groups (P > 0.05) (Figures 9E,F).

### 3.13 Effects of FZD2 overexpression on FZD2 and the Wnt5a/Ca^2+^/NFAT pathway in GC cells

**FIGURE 10:**
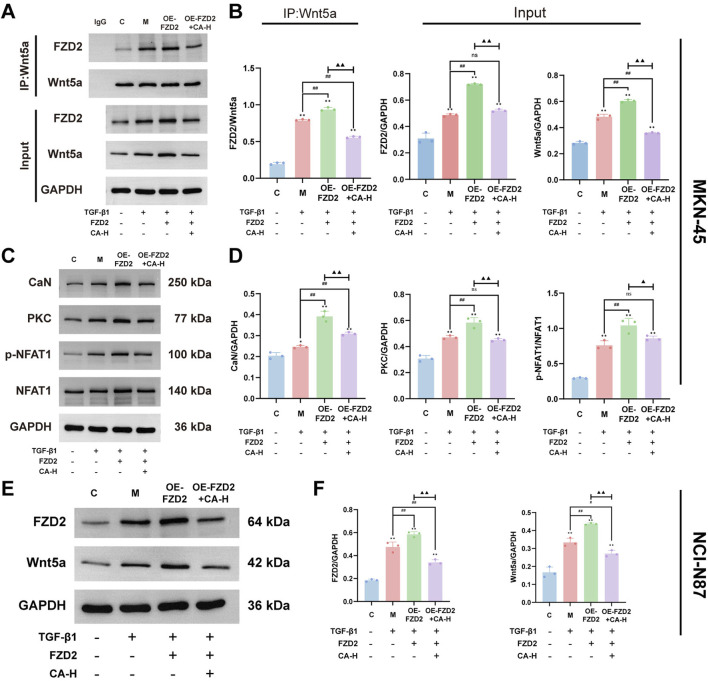
FZD2 mediates activation of the Wnt5a/Ca2+/NFAT signaling pathway. **(A,B)** Co-immunoprecipitation assay to detect the protein interaction between FZD2 and Wnt5a in MKN-45. **(C,D)** Western blot analysis of protein expression for CaN, PKC, and p-NFAT1/NFAT1 in MKN-45. **(E,F)** Western blot Analysis of FZD2 and Wnt5a Expression in NCI-N87 Cells. Compared with the group C, *p < 0.05, **p < 0.01. Compared with the group M, #p < 0.05, ##p < 0.01. Compared with the group OE-FZD2, ▲p < 0.05, ▲▲p < 0.01. ns, no significance.

The co-immunoprecipitation results showed that in MKN-45 cells after the TGF-β1-induced EMT model, the protein expression levels of FZD2 and Wnt5a increased. The interaction between the two proteins also increased (P < 0.01). After adenovirus transfection to overexpress FZD2 in the EMT model group, the expression of the two proteins and their interaction increased (P < 0.01). This suggests that there is interaction between FZD2 and Wnt5a and that regulating FZD2 affects the expression of Wnt5a. After CA intervention, the protein expression of FZD2 and Wnt5a as well as the interaction reduced (P < 0.01). These findings suggest that CA inhibits the expression of Wnt5a by inhibiting the expression of FZD2 (Figures 10A,B). Compared with the C group, the relative protein expression levels of CaN, PKC, and p-NFAT/NFAT in the M group increased (P < 0.05). Compared with the M group, FZD2 overexpression elevated the expression of CaN, PKC, and p-NFAT/NFAT (P < 0.01). Compared with the OE-FZD2 group, CA intervention reduced the levels of CaN, PKC, and p-NFAT/NFAT (P < 0.01). Additionally, Western blot was used to detect the protein expression of FZD2 and Wnt5a in NCI-N87 cells. The results showed that compared with the C group, the protein expression of FZD2 and Wnt5a in the M group increased (P < 0.01). After adenovirus transfection, the expression of FZD2 and Wnt5a increased compared to the M group (P < 0.01). After CA intervention, the expression of FZD2 and Wnt5a decreased compared to the OE-FZD2 group (P < 0.01). These results are consistent with those observed in MKN-45 cells.

## 4 Discussion

GC is a common global malignancy with high morbidity and mortality rates. The incidence of new cases exceeds one million annually, posing a significant public health concern. In recent years, natural products have emerged as valuable sources for drug discovery and development due to their structural diversity and biological activities and have provided promising therapeutic options for cancer treatment. Numerous studies have demonstrated the antitumor potential of natural products, which is mediated primarily through the inhibition of EMT, induction of various types of programmed cell death, and modulation of the tumor microenvironment (Chen et al., 2025). Cornus officinalis is a plant that contains a wide range of bioactive natural compounds and has recently attracted increasing scientific interest due to its potential therapeutic effects in various disease contexts. Among these compounds, CA, a polyphenolic constituent of Cornus officinalis, has been reported to have anticancer effects on several tumor types, including head and neck cancer, multiple myeloma, breast cancer, and osteosarcoma (Cortez et al., 2024). Although the anticancer potential of CA has been demonstrated in various cancer models, its mechanisms of action in GC remain insufficiently studied. In this study, we developed several machine learning models and integrated bioinformatics analysis, virtual screening, molecular dynamics simulations, and cellular experiments to comprehensively investigate the potential targets and mechanisms of CA in the treatment of GC.

In this study, we applied multiple machine learning algorithms to analyze GC datasets from public databases and identified FZD2 as a candidate gene of interest. As a member of the FZD receptor family, FZD2 has been reported to play a key regulatory role in the development and progression of various malignancies. The FZD family belongs to the classical G protein-coupled receptor (GPCR) superfamily, and its members are typically transmembrane proteins with highly conserved structural features. These proteins typically consist of an extracellular N-terminal domain, seven transmembrane α-helical segments, and an intracellular C-terminal domain. Among these regions, the N-terminal cysteine-rich domain (CRD) is a key structural unit that mediates specific binding to endogenous ligands, such as Wnt proteins, and is essential for signal transduction. Previous studies have shown that FZD family proteins are widely expressed in various malignant tumors and perform critical regulatory functions during tumor initiation and progression. For example, FZD10 promotes the self-renewal capacity, tumor-initiating potential, and metastatic ability of liver cancer stem cells by activating the β-catenin and YAP1 signaling pathways. Moreover, elevated expression of FZD10 has been shown to increase the proliferative activity of liver cancer stem cells, suggesting a potential role in maintaining cancer stemness (Wang et al., 2023). Additionally, aberrant overexpression of FZD6 in high-grade serous ovarian cancer (HGSOC) is significantly associated with reduced patient survival. Functional studies have demonstrated that FZD6 not only promotes rapid tumor growth and peritoneal metastasis in HGSOC but also induces chemoresistance to certain agents, thereby exacerbating tumor malignancy (Chen et al., 2024). FZD7, another highly expressed member of the FZD family in various cancers, can activate both β-catenin-dependent and β-catenin-independent Wnt signaling pathways, collectively promoting tumor growth and chemoresistance, suggesting its potential oncogenic role across multiple cancer types. Furthermore, differential gene expression analysis revealed that FZD2 is significantly upregulated in GC (Figure 3E). In addition, survival and diagnostic prediction models indicated that high FZD2 expression is not only significantly associated with poorer overall survival but also has strong predictive power for GC diagnosis (Figures 3F,G). Moreover, we performed validation of FZD2 using external datasets. The analysis revealed significant differences in FZD2 protein expression between normal tissues and GC tissues. Specifically, FZD2 expression was lower in normal tissues and higher in GC tissues (Figures 7A–E). *In vitro* experiments demonstrated that TGF-β1 stimulation in the model group promoted the migration and invasion of MKN-45 cells (Figures 8B–E), accompanied by an increase in FZD2 protein expression (Figures 9E,F). These findings suggest that elevated FZD2 expression may increase the invasiveness and metastatic potential of GC, thereby contributing to tumor progression.

Previous studies have indicated that FZD2 is closely associated with EMT, a hallmark of cancer cell acquisition of invasive and migratory capabilities, which is strongly linked to tumor metastasis and poor prognosis (Liu et al., 2024). Moreover, multiple studies have demonstrated that EMT plays a critical role in the initiation and progression of GC, primarily through signaling pathways such as the TGF-β, Wnt/β-catenin, and Hippo pathways, thereby promoting cell migration, invasion, immune evasion, and poor clinical outcomes (Luo et al., 2024; Hu et al., 2024). EMT refers to a biological process in which epithelial cells acquire mesenchymal-like properties by altering their adhesion, cytoskeletal organization, morphology, and motility. This process contributes significantly to multiple aspects of tumor progression, including invasion, metastasis, and immune evasion (Guo et al., 2025). Specifically, EMT endows epithelial tumor cells with enhanced motility and invasiveness, allowing them to detach from the primary site, invade surrounding tissues, and enter the bloodstream to form distant metastases (Malagoli et al., 2023). Previous studies have shown that FZD2 is involved in the regulation of EMT in epithelial cells and that its expression is associated with increased tumor invasiveness and metastatic potential (Gujral et al., 2014). In this study, we performed single-cell transcriptomic analysis to characterize the cellular composition and subpopulations within GCs and found that FZD2 expression was markedly enriched in epithelial cells (Figures 5A–E). The *in vitro* results revealed that E-cadherin expression was downregulated, whereas the expressions of N-cadherin, vimentin, and Snail were elevated in the model group, indicating that TGF-β1 stimulation may promote EMT-related phenotypic transformation in MKN-45 cells (Figures 9C,D). Notably, FZD2 protein expression was also significantly increased in the model group (Figures 9E,F), suggesting its involvement in EMT induction. These findings support the hypothesis that FZD2 may contribute to GC progression by acting as a potential regulator of tumor metastasis.

Immune infiltration analysis revealed a significant positive correlation between FZD2 expression and M2 macrophage abundance (Figure 4). M2 macrophages represent a major subtype of tumor-associated macrophages and are generally considered protumorigenic, playing key roles in the tumor microenvironment (Xiao et al., 2024). Previous studies have shown that M2 macrophages can induce EMT in epithelial cells by secreting various cytokines, thereby facilitating the shift from an adhesive epithelial phenotype to a more migratory mesenchymal phenotype (Liu et al., 2025). Moreover, EMT contributes to the enhancement of several malignant phenotypes, including increased cell migration and invasion, enhanced angiogenesis, increased resistance to chemotherapy, and increased immune evasion (Sumitomo et al., 2023). Taken together, the strong positive correlation between FZD2 expression and M2 macrophage infiltration, along with previous evidence highlighting the protumor functions of M2 macrophages in immune suppression and EMT induction, suggests that FZD2 may contribute to remodeling of the immune microenvironment in GC by modulating the polarization or functional state of M2 macrophages, thereby exerting potential tumor-promoting effects.

Furthermore, we constructed an oral bioavailability model based on Lipinski’s rule of five and Veber’s rule. Using this model, we identified a total of 160 core compounds that simultaneously satisfied both criteria (Figure 6A). Lipinski’s rule of five and Veber’s rule are important guidelines in the drug screening process, as they help predict the oral bioavailability of compounds and improve the drug likeness of candidate molecules, thereby facilitating more informed decision-making in drug design and development (Kumar et al., 2024). Subsequently, large-scale virtual screening combined with four machine learning-based toxicity prediction models identified CA as a potentially effective core compound for the treatment of GC (Figures 6B,C). To validate the predicted interaction between CA and FZD2, MD simulations were carried out, which further confirmed the structural stability and binding plausibility of the CA-FZD2 complex (Figures 6D–J). The results revealed that the RMSD of the protein backbone initially fluctuated but then gradually stabilized, remaining within the range of 0.3–0.5 nm, with a maximum deviation of approximately 0.85 nm. In contrast, the RMSD of the ligand relative to the protein backbone gradually increased during the first 20 ns and then fluctuated between 1.6 and 2.3 nm, suggesting dynamic movement and rearrangement within the binding pocket, without evidence of complete dissociation. The RMSF values of most protein residues were within the range of 0.1–0.4 nm, with certain regions reaching 0.5 nm, indicating the presence of flexible regions within the protein structure. Overall, no significant abnormal fluctuations were observed in the system. The Rg of the protein remained between 2.25 and 2.35 nm throughout the simulation, and the Rg components along the X, Y, and Z-axes showed no significant variation, indicating that the overall protein conformation remained compact and stable without structural relaxation or unfolding tendencies. Throughout the simulation, 1-8 hydrogen bonds were maintained between the protein and the ligand, with the majority of time points showing 4-7 hydrogen bonds, indicating relatively stable interactions between the two molecules. Finally, the free energy landscape plotted with RMSD and Rg as reaction coordinates revealed that the lowest free energy region was primarily concentrated in the area with an RMSD of 0.45–0.48 nm and an Rg of 2.27–2.30 nm, forming a single dominant energy minimum. In summary, the FZD2-CA complex exhibited favorable structural stability and binding characteristics during the simulation, supporting the stability and plausibility of the predicted binding conformation. *In vitro* experiments further supported a potential binding interaction between CA and FZD2, as both high and low concentrations of CA reduced FZD2 protein expression in the EMT model of MKN-45 cells (Figures 9E,F).

Moreover, KEGG pathway enrichment analysis revealed that the WNT signaling pathway was significantly enriched, suggesting its potential involvement in the biological processes relevant to the study (Figure 3D). The WNT signaling pathway, a highly conserved signaling cascade, plays important roles in cell proliferation, cancer initiation, and progression (Song et al., 2024). The WNT signaling pathway is mainly divided into the canonical Wnt/β-catenin pathway and the noncanonical Wnt/PCP and Wnt/Ca^2+^ pathways. The Wnt/Ca^2+^ signaling pathway primarily consists of Wnt ligands, Wnt receptors, G proteins, DVL, PLC, PKC, IP3, DAG, CaM, and CaMKII (Xue et al., 2025; Xie et al., 2025). Among the WNT family members, Wnt5a and Wnt11 are prominent ligands involved in noncanonical Wnt signaling. These ligands primarily interact with Frizzled receptors such as FZD2, and in certain contexts, their activity can be modulated by cofactors such as the RSPO2-LGR5 complex (Han et al., 2024). The interaction between FZD2 and the Wnt5a ligand is a well-characterized component of the Wnt/Ca^2+^ signaling pathway and plays a pivotal role in its activation (Martinez-Marin et al., 2025). This signaling axis has been implicated in various pathological processes, including cancer progression. During the activation of the Wnt/Ca^2+^ signaling pathway, the binding of FZD2 to its ligand Wnt5a initiates a cascade that activates the intracellular DVL and G proteins. These molecules synergistically stimulate PLC, which hydrolyzes phosphatidylinositol 4,5-bisphosphate to generate inositol 1,4,5-trisphosphate and DAG, two essential second messengers (Wang et al., 2024). IP3 subsequently binds to its receptor on the endoplasmic reticulum, promoting the release of Ca^2+^ into the cytoplasm and leading to a transient increase in the intracellular calcium concentration (Wang et al., 2025; Qin et al., 2024). In parallel, DAG activates PKC, which, in cooperation with elevated Ca^2+^ levels, contributes to the activation of the Ca^2+^/calmodulin-dependent protein CaMKII through phosphorylation. Moreover, excess cytosolic Ca^2+^ interacts with CaM to form the Ca^2+^/CaM complex, which further participates in the activation of transcription factors such as NFAT and NF-κB. These transcriptional events collectively modulate downstream gene expression programs involved in cellular proliferation, adhesion, and migration processes that are closely associated with tumor progression (Han et al., 2024). Therefore, this study further examined the expression of proteins involved in the Wnt5a/Ca^2+^/NFAT signaling pathway. In the EMT model of MKN-45 cells, the levels of Ca^2+^, Wnt5a, CaN, PKC, and p-NFAT/NFAT were markedly increased (Figure 9), suggesting that FZD2 may promote GC progression by modulating this pathway. Upon CA treatment, the expression of these proteins was suppressed to varying extents, indicating that CA may exert anti-GC effects by regulating FZD2 and subsequently inhibiting the Wnt5a/Ca^2+^/NFAT pathway (Figure 9). To further examine whether CA exerts its inhibitory effect through targeting FZD2, we employed adenoviral transfection to overexpress FZD2 in MKN-45 cells, followed by treatment with a high concentration of CA. Both co-immunoprecipitation and Western blot analyses demonstrated the interaction between FZD2 and Wnt5a and indicated that CA treatment reduced the activation of the Wnt5a/Ca^2+^/NFAT signaling pathway by suppressing FZD2 expression (Figures 10A–D). Consistently, CA treatment in NCI-N87 cells also reduced the expression of FZD2 and Wnt5a (Figures 10E,F), implying that the inhibitory effect of CA on FZD2 might have broader relevance across GC cell lines.

However, this study has several limitations. Although we have employed multiomics, ML, and MD to identify potential targets in GC and provide initial evidence for the modulatory effects of CA on FZD2 and the Wnt/Ca^2+^/NFAT pathway *in vitro*, further studies would help to fully confirm the direct regulatory relationship between FZD2 and this signaling cascade. In addition, considering the heterogeneity of tumors, we recognize that whether these findings apply to other GC cells requires further verification. Future studies incorporating additional omics data, more GC cells from different backgrounds, and further mechanism experiments are needed to help improve the reliability and translational potential of these findings.

## Data Availability

The datasets presented in this study can be found in online repositories. The names of the repository/repositories and accession number(s) can be found in the article/supplementary material.
